# Homogeneity versus diversity: inhibition of plasma PAI-1 in murine sepsis proved lethal in homogeneous cohorts but not in all-inclusive populations

**DOI:** 10.1186/cc11760

**Published:** 2012-11-14

**Authors:** P Raeven, KM Weixelbaumer, S Drechsler, A Klotz, M Jafarmadar, A Khadem, H Redl, S Bahrami, PJ Declerck, MF Osuchowski

**Affiliations:** 1 Ludwig Boltzmann Institute for Experimental and Clinical Traumatology, AUVA Trauma Research Center, Vienna, Austria; 2 Laboratory for Pharmaceutical Biology, KU Leuven, Belgium

## Background

Plasminogen activator inhibitor type 1 (PAI-1) inhibits fibrinolysis and its plasma increases correlate with exacerbated sepsis mortality. However, its exact role in abdominal sepsis outcomes is unclear. We aimed to test the effects of fibrinolysis restoration upon survival during acute polymicrobial sepsis using a custom-developed anti-PAI-1 monoclonal antibody. We specifically studied whether the effects of the treatment vary between all-inclusive populations and homogeneous cohorts stratified based on the risk of death.

## Methods

Mice underwent cecal ligation and puncture (CLP) and received a single intravenous injection of either anti-PAI-1 (10 mg/kg; MA-MP6H6) or control antibody (MA-Control). Three approaches were used: approach I, co-treatment (CLP and MA-MP6H6 administered at 0 hours) without stratification; approach II, post treatment (MA-MP6H6 at 18 hours post CLP) without stratification; and approach III, post treatment (MA-MP6H6 at 30 hours) with prospective stratification. Then 20 μl blood (facial vein) was sampled daily from all mice until day 5 and survival was followed for 28 days. In approach I, a group of mice was sampled at 6 hours and sacrificed at 24 hours to test the MA-MP6H6 efficacy. In approach III, mice were stratified into either predicted to survive (P-SUR) or die (P-DIE) based on circulating IL-6 measured at 24 hours post CLP.

## Results

MA-MP6H6 co-treatment blocked 60 to 100% of active plasma PAI-1 and restored fibrinolysis (fibrin plate assay) in septic animals at 24 hours post application. Without stratification, MA-MP6H6 co-treatment and 18-hour (also 30-hour) post treatment neither conferred benefit nor was detrimental. Post treatment with MA-MP6H6 after prospective IL-6 stratification at 14 ng/ml cutoff (48% sensitivity for P-DIE and 100% specificity for P-SUR) showed 15% exacerbation of mortality (*P *> 0.05). Cutoff readjustment to 3.3 ng/ml maximized P-DIE cohort homogeneity (82% sensitivity; 100% specificity) revealing harmful MA-MP6H6 effects: in P-SUR, no MA-Control-treated mice ever died, but 30% of MA-MP6H6-treated did (*P *< 0.05). In P-DIE, all MA-MP6H6-treated mice died within 72 hours, while MA-Control mice lived up to 8 days (*P *< 0.05). In co-treatment, MA-MP6H6 increased circulating IL-6 and CXCL-1/KC by threefold and twofold at 24 hours (MCP-1, MIP-1α, IL-1β, IL-10 and TNFα unaffected), but had no effect in post treatment (24 to 96 hours). Regardless of the treatment approach, MA-MP6H6 did not significantly alter hematological and/or organ function parameters.

## Conclusion

Restoration of fibrinolysis by neutralization of PAI-1 in acute sepsis lacks benefit and may prove fatal. Remarkably, the detrimental effect of MA-MP6H6 became apparent only after a maximally homogeneous separation of high/low risk-of-death cohorts. A prospective separation of septic patients into various well-defined, homogenous cohorts is critical in revealing negative side effects of tested anti-sepsis therapeutics.

